# Self-compassion modulates autonomic and psychological responses to stress among adults with generalized anxiety disorders

**DOI:** 10.3389/fpsyt.2025.1461758

**Published:** 2025-03-14

**Authors:** Lijun Sun, Xuejun Qi, Xi Luo, Ying Wang, Xianwei Che, Yonghui Shen

**Affiliations:** ^1^ Affiliated Mental Health Center & Hangzhou Seventh People’s Hospital, Zhejiang University School of Medicine, Hangzhou, China; ^2^ School of Nursing, Hangzhou Medical College, Hangzhou, China; ^3^ Centre for Cognition and Brain Disorders, The Affiliated Hospital of Hangzhou Normal University, Hangzhou, China

**Keywords:** self-compassion, generalized anxiety disorder, heart rate variability, stress, GAD

## Abstract

**Background:**

Self-compassion is associated with emotional well-being, yet its benefits in generalized anxiety disorder (GAD) patients remain unclear. This study investigated the impact of self-compassion on emotional and physiological stress responses in individuals diagnosed with GAD.

**Methods:**

Seventy-seven GAD patients were categorized into high (n = 39) and low (n = 38) self-compassion groups using the Self-Compassion Scale. Electrocardiograms were recorded during a stress-inducing task, in which negative feedback was provided on personal intelligence and career development. Participants reported state anxiety and perceived stress pre- and post-task.

**Results:**

When exposed to a stressor, individuals with higher self-compassion had lower heart rates (*t*_(75)_ = -2.06, *p* = 0.043), higher heart rate variability (*t*_(75)_ = 2.73, *p* = 0.04), and lower anxiety (*t*_(75)_ = -2.07, *p* = 0.041) compared to the lower self-compassion group. Moreover, heart rate variability was negatively correlated with anxiety across patients (*r* = -0.31, df = 75, *p* = 0.03).

**Conclusion:**

These results highlight the role of self-compassion in managing psychological and physiological responses to stress in GAD patients and indicate the potential of self-compassion interventions in GAD treatments.

## Introduction

Generalized anxiety disorder (GAD), a prevalent and debilitating mental condition, is defined by persistent anxiety, uncontrollable worry, and often, heightened autonomic arousal and emotional dysregulation (1, 2). GAD patients frequently exhibit increased sympathetic nervous system activity, reduced parasympathetic tone and impaired heart rate variability (HRV), a key indicator of autonomic flexibility and emotional resilience (3, 4). These physiological disruptions are often linked to abnormal hypothalamic-pituitary-adrenal (HPA) axis activity, which contributes to a hypersympathetic state and chronic stress responses (4, 5). Given the profound impact of autonomic dysregulation on emotional well-being in GAD, identifying protective factors that can mitigate these physiological and psychological stress responses is very important.

Self-compassion, an approach entailing kindness and care toward oneself during adversities (6), has emerged as a pivotal factor for promoting emotional and physiological resilience. Research consistently demonstrates that self-compassion is positively associated with emotional well-being (7, 8) and negatively linked to life stress and negative emotional states (9, 10). Moreover, self-compassion has been shown to buffer against the effects of both daily stressors (11, 12), and acute stress induced in laboratory settings (13, 14). These findings highlight the potential of self-compassion as a protective factor in stress regulation.

At the physiological level, self-compassion has been associated with enhanced vagus nerve-mediated HRV, a marker of autonomic flexibility and emotional regulation (15, 16). HRV reflects the dynamic interplay between the sympathetic and parasympathetic nervous systems and is considered a key indicator of overall health and the mind-body connection (17, 18). Individuals with higher levels of self-compassion tend to exhibit greater HRV, suggesting improved physiological and psychological adaptability to stress (16). However, despite these promising findings, the majority of studies investigating the relationship between self-compassion and HRV have focused on healthy populations (19–21), leaving a significant gap in understanding how self-compassion influences autonomic regulation in clinical populations, particularly those with GAD.

Individuals with GAD often report lower levels of self-compassion compared to healthy individuals, which may exacerbate their emotional and physiological stress responses (22, 23). Nevertheless, emerging evidence suggests that self-compassion interventions can improve emotional regulation and reduce anxiety and depression symptoms in GAD patients (23, 24). Self-compassion is thought to activate a soothing function that inhibits the threat-defensive response triggered by stress or anxiety (25). However, the specific mechanisms through which self-compassion influences psychophysiological responses, such as HRV, in individuals with GAD remain poorly understood. This represents a critical gap, as understanding these mechanisms could inform the development of targeted interventions to improve both psychological and physiological outcomes in GAD.

To address this gap, this study examined the relationship between self-compassion and HRV during a stress-inducing task in individuals with GAD. The Self-compassion Scale (SCS) can assess trait levels of the general inclination to respond self-compassionately during instances of failure, or life challenges (26). Eligible GAD patients were categorized into high and low self-compassion groups (High SC and Low SC) based on their SCS scores. A Stress-inducing task, adapted from the Raven Standard Reasoning Test, was used to elicit physiological and psychological stress responses by providing negative feedback on personal intelligence and career development (27–29). We hypothesized that the High SC group would exhibit higher HRV levels and lower anxiety, and perceived stress levels compared to the Low SC group when confronted with stress. By exploring the interplay between self-compassion and autonomic regulation in GAD, this study would provide new insights into the potential therapeutic benefits of self-compassion-based interventions for this population.

## Materials and methods

### Participants

Participants in this study were part of a clinical trial designed to investigate the effect of a self-compassion intervention and mindfulness training compared to a treat-as-usual group in adults with GAD (24). The data utilized in this study were collected at baseline, prior to the commencement of any interventions. We recruited participants from patients at the Hangzhou Seventh People’s Hospital through advertisement posters and flyers, inviting individuals with GAD symptoms to participate in the study. Trained clinicians conducted a DSM-5 principal diagnostic evaluation using Mini-International Neuropsychiatric Interview (M.I.N.I.) (1, 30). Inclusion criteria for the study were: (i) adults aged 18 to 65; (ii) a primary diagnosis of GAD with a Hamilton Anxiety Rating Scale score of 14 or higher; (iii) a Hamilton Depression Rating Scale score of less than 23; and (iv) at least a secondary education level. Exclusion criteria included: (i) alcohol or substance use disorder; (ii) psychiatric and medical comorbidities requiring treatment (e.g., bipolar disorder, schizophrenia, suicidal ideation or risk); (iii) conditions severely limiting participation (e.g., severe physical disease, cognitive dysfunction, hearing impairment); (iv) being pregnant or breastfeeding; and (v) current involvement in other psychotherapy. All participants gave informed written consent before beginning the study and all study procedure were approved by the ethics committee in the Hangzhou Seventh People’s Hospital (2021067). A total of 77 GAD patients were categorized into high (n = 39) and low (n = 38) self-compassion group using the Self-Compassion Scale. The median split was used to divide participants into High SC (above the median) and Low SC (below the median) groups, an approach consistent with previous studies (21, 31). Two groups did not differ in gender, course of disease, or years of education. Individuals with high SC had lower levels of depression and anxiety than those of low SC (**Table 1**).

**Table 1:** Demographics and baseline psychological measures of the High SC and Low SC groups.

	Total (*n* = 77)	High SC (*n* = 39)	Low SC (*n* = 38)	*p* value^a^
Age, years: mean (SD)	37.39 (12.81)	41.03 (12.51)	33.66 (12.17)	0.01^*^
Gender, n (%) Female Male	53 (68.8) 24 (31.2)	25 (64.1) 14 (35.9)	28 (73.7) 10 (26.3)	0.36
Marital Status, n (%) Married Single/Separated	49 (63.6) 28 (36.4)	31 (79.5) 8 (20.5)	18 (47.4) 20 (52.6)	0.003^**^
Education, n (%) Secondary High school University degree Postgraduate degree	18 (23.4) 16 (20.8) 38 (49.4) 5 (6.5)	9 (23.1) 12 (30.8) 17 (43.6) 1 (2.6)	9 (23.7) 4 (10.5) 21 (55.3) 4 (10.5)	0.10
Employment, n (%) Unemployed/Housewife/Retired Employed	18 (23.4) 59 (76.6)	12 (30.8) 27 (69.2)	6 (15.8) 32 (84.2)	0.12
Duration of patients had general anxiety, years, n (%) 0.5-1 1-3 3-5 5-10 ≥10	25 (32.5) 20 (26) 11 (14.3) 11 (14.3) 10 (13)	12 (30.8) 12 (30.8) 4 (10.3) 4 (10.3) 7 (17.9)	13 (34.2) 8 (21.1) 7 (18.4) 7 (18.4) 3 (7.9)	0.40
SCS score, mean (SD)	2.83 (0.62)	3.29 (0.37)	2.36 (0.44)	0.00^***^
HAMA score, mean (SD)	22.71 (5.97)	20.79 (5.85)	24.68 (5.49)	0.004^**^
HAMD score, mean (SD)	15.12 (4.79)	13.97 (5.27)	16.29 (3.98)	0.03^*^

High SC, High self-compassion group; Low SC, Low self-compassion group; SCS, Self-compassion Scale; HAMA, Hamilton Anxiety Rating Scale; HAMD, Hamilton Depression Rating Scale; *denotes *P* < 0.05; **denotes *P* < 0.01; ***denotes *P* < 0.001.

a: Estimated by χ2 test for categorical variables, and independent t-tests for continuous variables.

### Design and procedure

**Figure 1:**
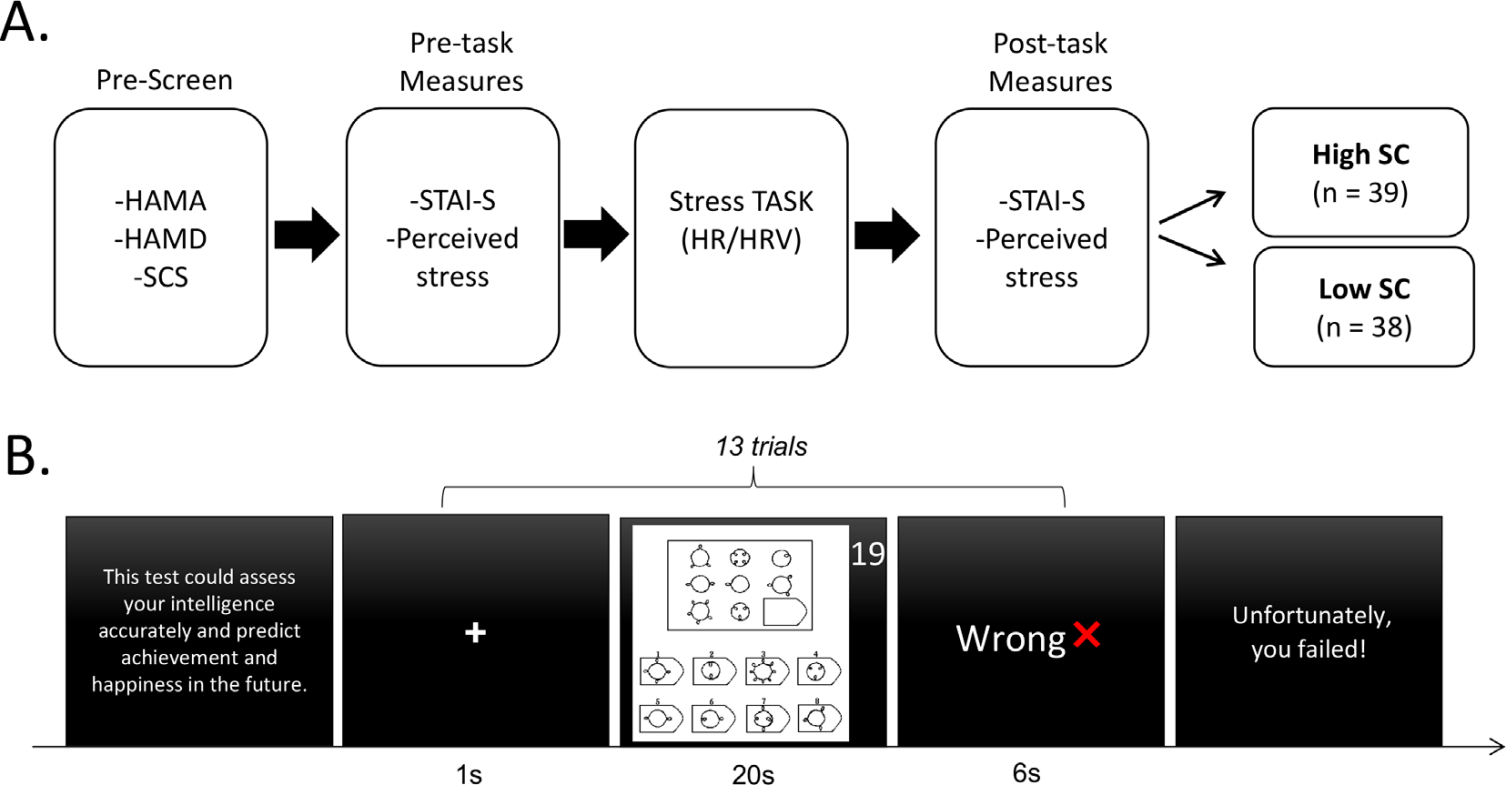
Study design and procedure. **(A)** Experimental procedure of this study. **(B)** Schematic diagram of the Stress-inducing task. HAMA, Hamilton Anxiety Rating Scale; HAMD, Hamilton Depression Rating Scale; SCS, Self-compassion Scale; HR, heart rate; HRV, heart rate variability; STAI-S, State form of Spielberger’s State-Trait Anxiety Inventory; High SC, high self-compassion group; Low SC, low self-compassion group.

After the clinical interview, eligible participates were first completed the Self-Compassion Scale (32), the State form of Spielberger’s State-Trait Anxiety Inventory (33) and self-reported Perceived stress. Then they were set up for the electrocardiograph (ECG) recording. Participants then underwent the Stress-inducing Task (27, 28). At the completion of the Stress-inducing Task, participants completed the second STAI-S and self-reported Perceived stress (**Figure 1A**). Participants were then categorized into High SC or Low SC group.

For the Stress-inducing task, 13 difficult items from the Raven Standard Reasoning Test (Chinese City Edition) were selected to induce pressure, specifically items B12, C10, C12, D9, D10, D11, D12, E7, E8, E9, E10, E11 and E12 (27, 28). At the beginning of the Stress-inducing task, participants were informed that this test could assess one’s intelligence accurately and predict achievement and happiness in the future. The task consisted of 13 trials and each trial started with a fixation (1 s), followed by the presentation of a reasoning item with 20-second countdown, during which participants were asked to think and answer. These questions are so difficult that almost no one can answer them correctly in such a short time. When the countdown is over, the algorithm would display feedback on their answers for 6 seconds, among which two questions were given ‘Correct’ randomly and the rest were given ‘Wrong’ as negative feedback. At the end of the task, the computer screen presented one sentence, ‘Unfortunately, you failed!’ (**Figure 1B**). It is worth noting that two groups had no difference in education, which could have an impact on the performance of the task.

### Measures

#### Hamilton anxiety rating scale

This is a well-validated instrument used by clinicians to assess the severity of anxiety symptoms (34). It contains 14 items, and each item score ranges from zero (no symptoms) to 4 (the worst severity symptom). The total score ranges from 0 to 56. Scores ≤ 7 were considered no anxiety and 8-14 = mild anxiety; 15-23 = moderate; scores ≥ 24 indicate severe (35). It is well-validated for the Chinese population (36).

#### Hamilton depression rating scale

This is a clinician-rated scale developed to evaluate depressive symptoms (37). This scale comprises 17 items, all rating from zero (no symptoms) to 4 (the worst symptom severity). The Chinese version has excellent interrater reliability and good validity (38) and the total score ranges from 0 to 52. The severity ranges were no depression (0-7); mild depression (8-16); moderate depression (17-23); and severe depression (≥ 24) (39).

#### Self-compassion scale

This is a 26-item self-reported scale designed to measure individual differences in self-compassion (32). The use of the SCS as a total score representing overall self-compassion was supported across cultures (40, 41). Items are rating scores vary from 1 (almost never) to 5 (almost always). The total mean is used on a five-point scale. The Chinese version has shown good reliability and validity (42). In the current study, the level of self-compassion ranged from 1.33 to 4.45, with a mean level of 2.83 (SD = 0.62). The SCS was reported to have a Cronbach’s alpha of 0.83 and test–retest reliability of 0.89 (42).

#### State form of Spielberger’s state-trait anxiety inventory

This is a commonly used self-reported scale with 20 items that measure state anxiety (33). All items are rated on a four-point scale ranging from 1 (not at all) to 4 (very much so). Total score ranges from 20 to 80 and higher scores indicate greater anxiety. Chinese version is well-validated (43).

#### Perceived stress

Participants reported how much stress they are experiencing currently using a 10-point Likert scale from 1 (almost no) to 10 (almost worst) (44).

### ECG recording

ECG was collected using a BITalino (r)evolution Board Kit BT (BITalino, Portugal) (http://bitalino.com/en/). Three Ag/AgCI electrodes were used, two of which were placed in the chest near the clavicles bilaterally and one electrode was placed at the lower edge of the left rib cage. ECG data can be captured and recorded through the OpenSignals (r)evolution software (v.2017, BITalino, Portugal) on a separate computer, with a sampling rate of 1000Hz.

### Data analysis

For the ECG data, inter-beat-interval (IBI) series were derived by Pan-Tompkins algorithm that identifies the peak of the R wave as the fiducial point (45). Artefacts were visually checked and edited if necessary according to published guidelines (46). IBI series were then transformed to beat-per-minute (BPM) series. Continues data were segmented based on the onset of the feedback (-1 to 6 s). Trials during the feedback of ‘Wrong’ were kept and baseline corrected for each trial (-1- 0 second, where time 0 represents the onset of the feedback). This method is believed to control for individual differences in baseline heart rate and to capture the dynamics of event-related heart rate change in a short period (47). Heart rate data were then averaged across trials for each participant, and area under the curve (AUC) was calculated with the linear trapezoidal rule to measure event-related heart rate change during the presentation of negative feedback.

After identified and checked the IBI, R-R-Interval were derived and linearly interpolated to 4 Hz to get evenly sampled signals (48). Interpolated R-R-Interval wave was then detrended through a high-pass filter with the cutoff frequency of 0.02 Hz (49). We employed a time-varying autoregressive (TVAR) model for the time-frequency analysis of HRV (50, 51). Particularly, the TVAR model has the advantage of providing smooth spectral components and accurate estimation of the power spectrum (50, 51). It has already been used for the investigation of beat-to-beat spectra during cold pain stress (15). The model order was set to 12 according to the literature (50). The HRV parameters nHF (normalized high frequency) was calculated over time for each subject and nHF was expressed as the relative value of high frequency component (0.15-0.4 Hz) in proportion to the total power minus the very low frequency component (0 -0.04 Hz) (52). The nHF-HRV generally reflects the balance of the sympathetic and the parasympathetic nerve activities (52). Trials during the feedback of ‘Wrong’ (-1 to 6 s) were analyzed and baseline corrected for each trial (-1- 0 second, where time 0 represents the onset of the feedback).

We then assessed the differences in heart rate change between the High SC and the Low SC groups. Due to the dynamic nature of heart rate, we focused on identifying specific time windows where these differences would be significant. To achieve this, we employed a sliding window approach with a step size of 50 ms and 500 ms as window size. Within each window, heart rate change was computed as the difference of AUC between the High SC and the Low SC groups (53). Pearson correlations were subsequently computed to examine the relationship of heart rate change and anxiety.

### Statistics

Using SPSS (version 23; IBM Corp, Armonk, NY), the initial comparison of baseline demographic and clinical characteristics between groups was conducted using independent sample *t*-tests for continuous variables and χ2 tests for categorical variables. Group differences between the High SC and Low SC groups in changes in state anxiety and perceived stress after the stress-inducing task were then examined using independent sample *t*-tests. Additionally, independent *t*-tests were performed on the changes in heart rate and nHF-HRV of the two groups respectively to investigate the physiological change. A series of Pearson correlation analyses were also conducted between heart rate changes and behavior changes to explore the body-mind connection.

## Results

### Demographic and descriptive analysis

**Figure 2:**
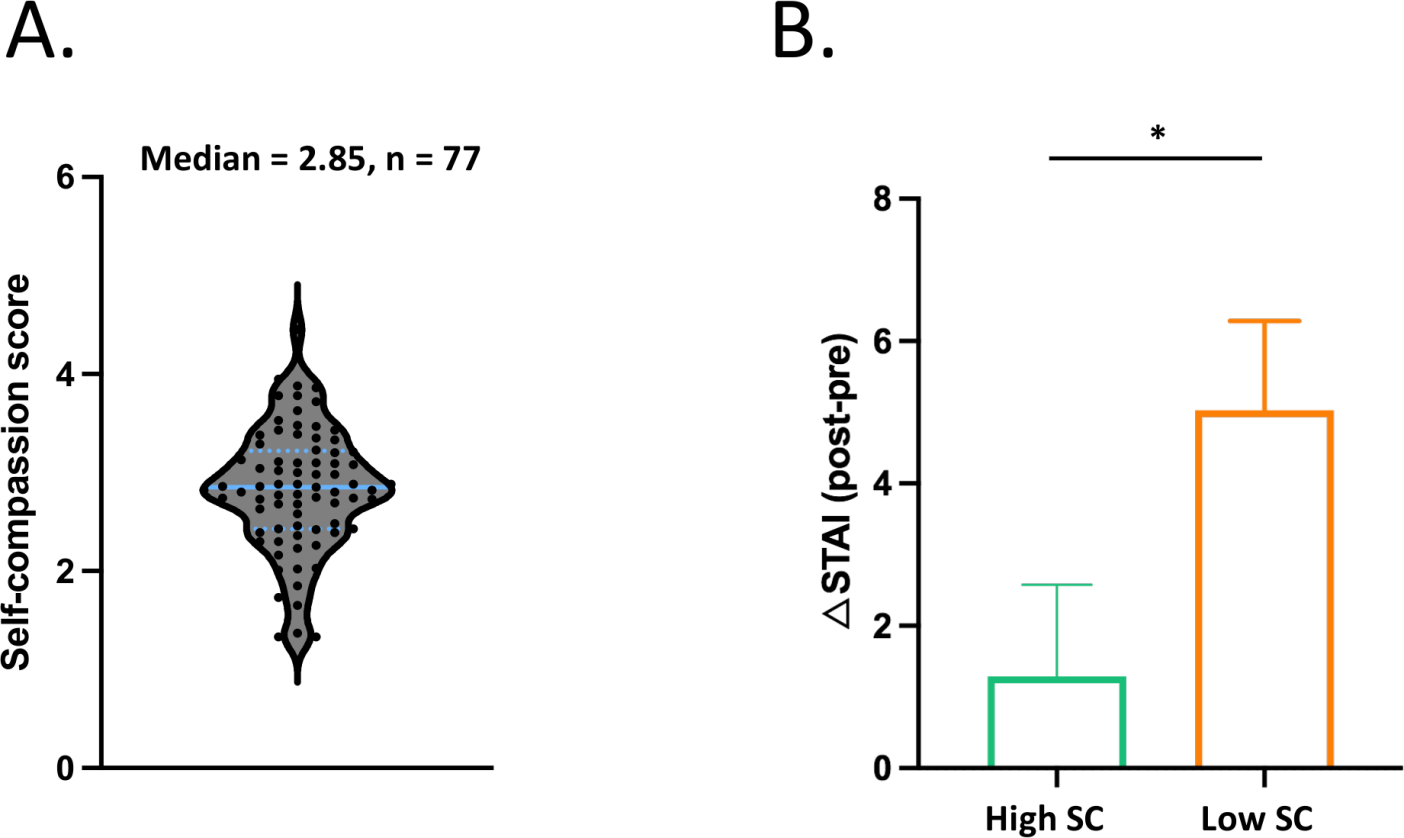
Distribution of self-compassion scores and group difference in changes in state anxiety. **(A)** Participants were divided into High SC (n = 39) and Low SC (n = 38) groups based on their self-reported self-compassion score (Median = 2.85). **(B)** The results show a significant group difference in changes in state anxiety (*t _(75)_* = -2.07, *p* = 0.041). The asterisk indicates statistical significance (*p* < 0.05). The column and error bar represent the mean and the standard error of the mean, respectively.

Participants were split into High SC and Low SC according to the median (Median = 2.85) of the self-reported self-compassion score (**Figure 2A**). T-tests revealed that the associations between High SC and Low SC were significantly different on self-compassion (*t _(75)_* = 10.11, *p* < 0.001), anxiety (*t _(75)_* = -3.00, *p* < 0.01) and depressive symptoms (*t _(75)_* = -2.17, *p* < 0.05) (**Table 1**).

### State Anxiety and Perceived Stress

Independent *t*-test showed that High SC and Low SC were significantly different on the changes of state anxiety (*t _(75)_* = -2.07, *p* = 0.041, Cohen’s d = 0.47). Individuals in High SC group reported less state anxiety after Stress-inducing task (**Figure 2B**). There was no group difference on the changes of the perceived stress (*p* = 0.55).

### Heart rate

**Figure 3:**
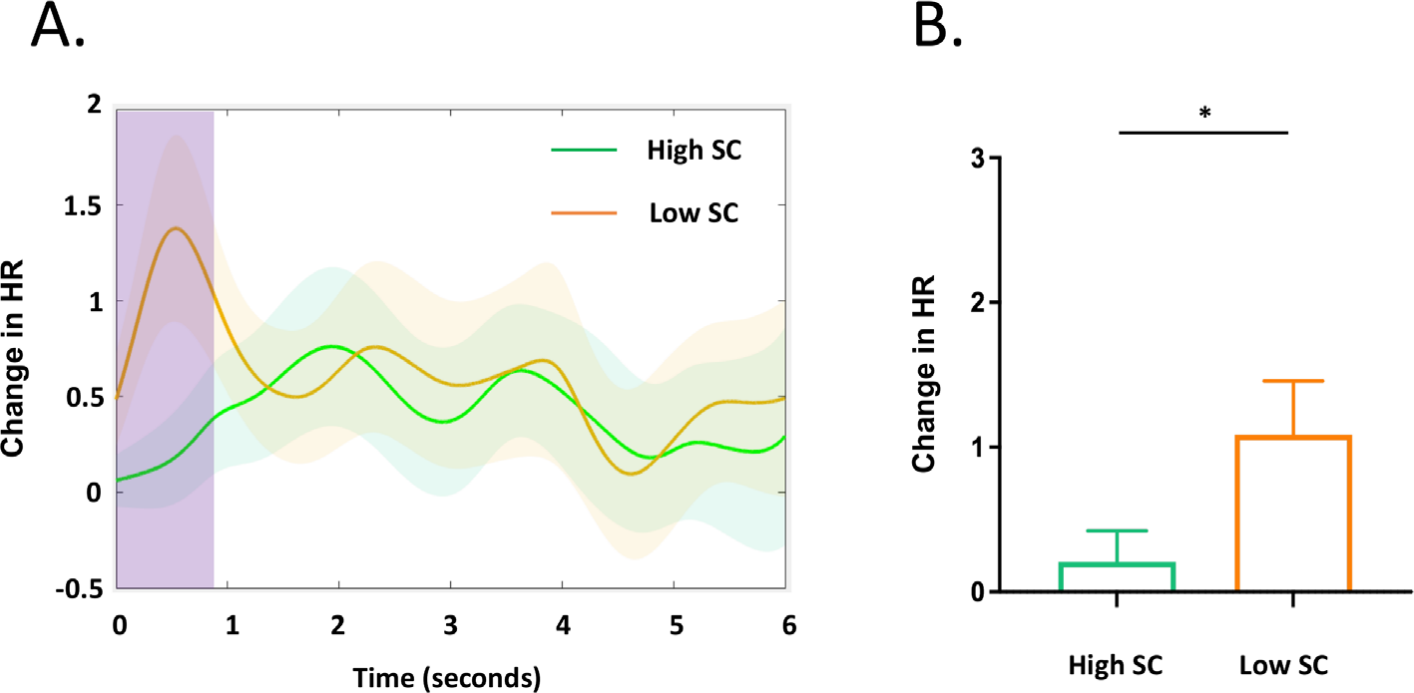
Heart rate response during negative feedback. **(A)** The pink column highlights the time window (0 - 0.85s) during which there is a group difference in HR change between the High SC and Low SC group. The background colors indicate the 95% confidence of intervals. **(B)** Individuals with high SC group had lower heart rate response to the negative feedback than the Low SC group (*t _(75)_* = -2.25, *p* = 0.03). The asterisk indicates statistical significance (*p* < 0.05).

Independent *t*-test found that High SC group had lower heart rate change than Low SC group (*t _(75)_* = -2.25, *p* = 0.03, Cohen’s d = 0.52) during negative feedback (0 - 0.85 s) (**Figure 3**).

### Heart rate variability

**Figure 4:**
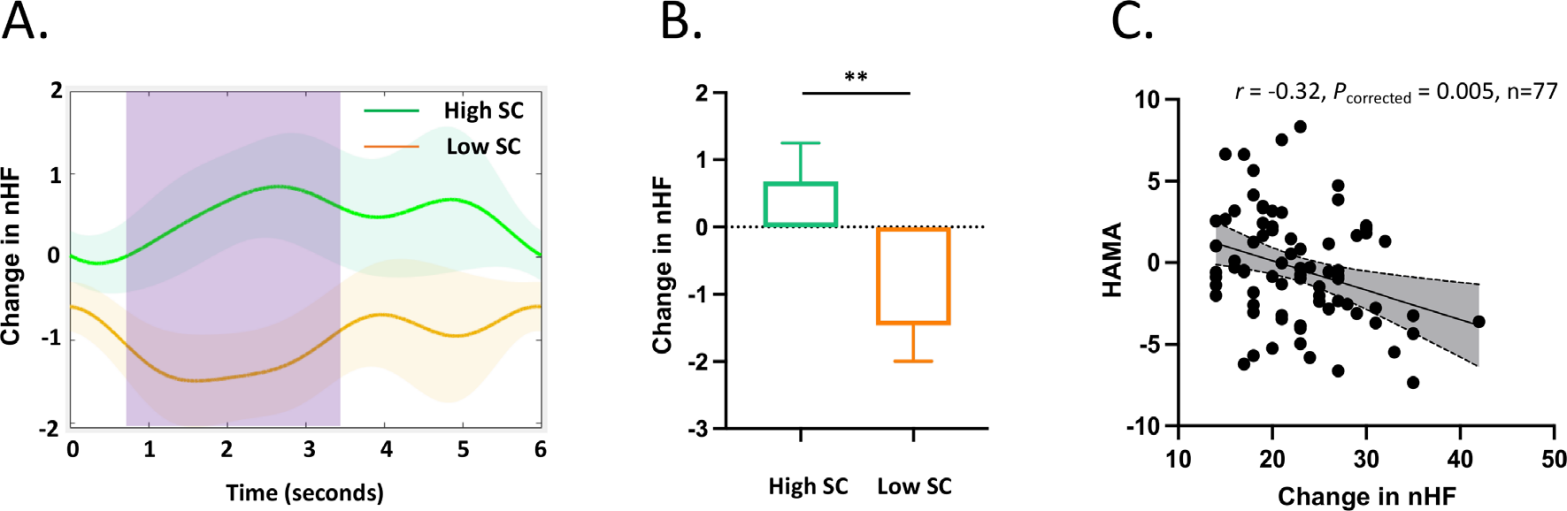
Changes in HF-HRV during negative feedback. **(A)** There is a significant difference in the HF-HRV between the High SC and the Low SC in the time window of 0.7 - 3.4 seconds, indicated by the pink column. **(B)** The high SC group had a higher HF-HRV change compared to the Low SC group (*t _(75)_* = 2.64, *p* = 0.01). **(C)** HF-HRV was negatively correlated with HAMA anxiety (*r* = - 0.32, *df* = 75, *p* = 0.005).

As shown in the **Figure 4**, the independent *t*-test revealed that High SC group showed higher nHF-HRV than Low SC group during 0.7 - 3.4s (*t _(75)_* = 2.64, *p* = 0.01, Cohen’s d = 0.60), indicating more adaptive response to stress.

### Correlation analyses

Increased HF-HRV was associated with lower HAMA in all data pool (*r* = - 0.32, *df* = 75, *p* = 0.005). The correlations among variables were also presented (see Supplementary material, **Supplementary Table S1**).

## Discussion

Previous research has demonstrated the stress-buffering effects of self-compassion among healthy populations. This study investigated the emotional protective effect of self-compassion among individuals diagnosed with GAD by examining both subjective reports and physiological responses measuring with heart rate and heart rate variability. Results showed that the high self-compassion group exhibited superior emotional well-being and diminished physiological stress responses. Specifically, those with higher levels of self-compassion reported reduced anxiety levels, lower heart rates, and increased heart rate variability after experiencing a stress-inducing task. Additionally, anxiety levels measured by the HAMA were significantly negatively correlated with heart rate variability.

Following the stress-inducing task, individuals in the high self-compassion group demonstrated lower anxiety levels compared to those in the low self-compassion group. This result supports our hypothesis that self-compassion plays an important role in fostering emotional well-being, extending its effect from healthy people to anxious patients confronting stress (19, 21). However, no difference was observed in the subjective stress between the two groups. This discrepancy between subjective reports and physiological measures (e.g., HRV) may reflect distinct pathways through which self-compassion operates, buffering autonomic arousal without immediately altering subjective stress perceptions. Alternatively, methodological limitations, such as the use of a single-item stress measure, may lack the sensitivity to capture subtle changes in perceived stress. Future studies could employ multi-dimensional stress scales or ecological momentary assessment to better understand the relationship between physiological and subjective stress responses (54, 55).

In one way, state anxiety was evaluated with a well-established measurement of STAI-T (33). Meanwhile, a single question was used to assess perceived stress in this study which may lack sensitivity and reliability. In another way, GAD individuals could be more sensitive to anxiety feelings than perceived stress in these negative scenarios. In either case, more studies are needed to clarify the inconsistent findings.

Heart rate analyses revealed significant group differences at the early stage of negative feedback (0 - 0.85 s). Individuals in the low self-compassion group exhibited significantly higher heart rates compared to those in the high self-compassion group. This result implies that when confronted with adverse feedback, the high self-compassion group showed less nervousness. This is consistent with previous evidence that self-compassion activates soothing and satisfaction systems, downregulates threat and drive systems, leads to parasympathetic activation, and decreases in physiological arousal, such as decreased heart rate and increased heart rate variability (56–58).

Our findings further provide mechanistic insights into the effects of self-compassion on anxiety. In line with the literature, this study found that the high self-compassion group exhibited increased heart rate variability (16, 19), indicating more efficient emotional regulation (59), enhanced cognitive processing of emotional information (60), as well as implications for both physical and psychological health (18, 61). Interestingly, the high self-compassion group demonstrated increased HRV at 0.7-3.4 seconds, which was after the early heart rate arousal to negative feedback. These dynamic changes in heart rate and HRV suggest the sympathetic arousal as well as the regulatory control over the system. Indeed, HRV serves as a potential marker of medial prefrontal cortex activation, guiding brain regions such as the amygdala and brainstem that influence cardiac regulation, thus reflecting the process of emotional regulation (18, 62). One recent study has further proposed a causal relationship between DLPFC and self-compassion in the context of social distress with brain stimulation technology (63). Moreover, a line of research has targeted the frontal-vagal network to treat affective disorders such as depression (64, 65). Building on these studies, our data demonstrate for GAD individuals, that the role of self-compassion in modulating the frontal-vagal circuitry implicated in emotion regulation and bodily arousal.

This novel mechanistic evidence is corroborated by the correlation between changes in HF-HRV and anxiety. When the two groups were pooled together, increased HF-HRV was associated with less anxiety (p = 0.005). This finding further highlights the role of HF-HRV in the regulatory control over sympathetic systems as well as the translation to emotions. It is also consistent with the findings that self-compassion fosters significant adaptability to physiological and psychological responses to emotions (16).

The findings have clinical significance. Firstly, self-compassion, serving as an adaptive emotion regulation tool, predicts flexible physiological responses to stress. Encouraging self-compassion in anxiety disorder patients may help in more flexible emotional and physiological adjustments during stressful events. In fact, intervention and treatment programs focusing on nurturing self-compassion, like Mindful Self-compassion (66) and Compassion-focused therapy (67), is becoming prevalent within clinical practice. Furthermore, enhancing self-compassion could positively impact both mental and physical health by improving heart rate variability and parasympathetic activation, which is crucial factors in cardiovascular health.

This study has limitations. First, while it establishes a correlation between self-compassion and reduced stress responses in GAD, it does not demonstrate causality. Future research should employ experimental designs, such as inducing self-compassionate states or implementing self-compassion interventions, to explore causal relationships. Second, the inconsistency between subjective stress measures and physiological responses warrants further investigation. Additionally, self-reported levels of self-compassion may be susceptible to reporting biases. Future studies could induce a self-compassionate state or introduce self-compassion interventions to draw causal inferences. Moreover, ethical prevented medication discontinuation, potentially influencing the results. Investigating long-term interventions’ impact on heart rate variability could offer valuable insights into using it as an assessment tool. Furthermore, exploring the heart-brain covariation mechanism during self-compassion may enhance understanding of the protective effect (68). Finally, while self-compassion is clearly a protective factor, its impact should not be overstated. Other variables, such as depression and anxiety, may also play significant roles. Future studies should carefully control for these covariates to clarify their influence.

In conclusion, this study investigated the impact of self-compassion on psychological and physiological responses in GAD patients. Our results revealed that higher self-compassion levels correlated with lowered heart rate, increased heart rate variability, and reduced anxiety during negative feedback. Importantly, heart rate variability exhibited a significant negative correlation with anxiety levels measured by HAMA. These findings highlight the adaptability of self-compassion in helping GAD patients manage psychological and physiological stress reactions, though further research is needed to establish causality and clarify the mechanisms underlying these effects.

## Data Availability

The raw data supporting the conclusions of this article will be made available by the authors, without undue reservation.
