# Supplementary material for: Self-compassion modulates autonomic and psychological responses to stress among adults with generalized anxiety disorders
Source: Front Psychiatry. 2025 Mar 14;16:1461758. doi: 10.3389/fpsyt.2025.1461758 (PMC11949995; doi:10.3389/fpsyt.2025.1461758)
Supplement: Supplementary file 1 [file DataSheet1.pdf]

Supplementary Material

1 Supplementary Tables

Table S1. Correlation coefficients of variables in all patients

Variables	1	2	3	4	5	6	7	8	9	10	11
1. HAMA	-										
2. HAMD	0.61**	-									
3. SCS	-0.30**	-0.29**	-								
4. STAI_Pre	0.38**	0.40**	-0.53*	-							
5. STAI_Post	0.35**	0.47**	-0.57**	0.70**	-						
6. changes in STAI (Post-Pre)	0.01	0.10	-0.16	-0.31**	0.41**	-					
7. PS_Pre	0.17	0.16	-0.40**	0.67**	0.53**	-0.15	-				
8. PS_Post	0.26*	0.33**	-0.43**	0.48**	0.73**	0.32**	0.68**	-			
9. changes in PS (Post-Pre)	0.12	0.21	-0.05	-0.25*	0.25*	0.59**	-0.37**	0.43**	-		
10. changes in HR	0.19	0.21	-0.12	0.10	0.13	0.03	0.04	0.07	0.03	-	
11. changes in HRV	-0.32**	-0.26*	0.21	-0.16	-0.08	0.00	0.00	0.11	0.13	-0.13	-
